# Potential Role of SWI/SNF Complex Subunit Actin-Like Protein 6A in Cervical Cancer

**DOI:** 10.3389/fonc.2021.724832

**Published:** 2021-07-29

**Authors:** Qingying Wang, Zuozeng Cao, Yingze Wei, Jiawen Zhang, Zhongping Cheng

**Affiliations:** ^1^Department of Obstetrics and Gynecology, Shanghai Tenth People’s Hospital, School of Medicine, Tongji University, Shanghai, China; ^2^Department of Obstetrics and Gynecology, Xinhua Hospital Chongming Branch, Shanghai Jiao Tong University School of Medicine, Shanghai, China; ^3^Department of Pathology, Nantong Tumor Hospital, Nantong, China; ^4^Department of Obstetrics and Gynecology, Shanghai General Hospital, Shanghai Jiao Tong University, Shanghai, China; ^5^Reproductive Medicine Center, Department of Obstetrics and Gynecology, Shanghai Jiao Tong University, Shanghai, China

**Keywords:** cervical cancer, ACTL6A, proliferation, cell cycle, GSEA

## Abstract

SWI/SNF complex subunit Actin-like protein 6A (ACTL6A) has been regarded as an oncogene, regulating the proliferation, migration and invasion of cancer cells. However, the expression pattern and biological role of ACTL6A in cervical cancer have not been reported. In this study, the mRNA expression and protein level of ACTL6A in cervical cancer samples were determined by public database and immunohistochemical (IHC) analysis. The effects of ACTL6A on cervical cancer cells were investigated *via* MTT, colony-formation assay, tumor xenografts and flow cytometry. Gene set enrichment analysis (GSEA) was used to explore the potential mechanism of ACTL6A in regulating tumorigenesis of cervical cancer. The results revealed that ACTL6A was markedly upregulated in cervical cancer tissues. Silencing ACTL6A expression resulted in decreased cervical cancer cell proliferation, colony formation and tumorigenesis *in vitro* and *in vivo*. Furthermore, we demonstrated that knockdown of ACTL6A induced cell cycle arrest at G1 phase, ACTL6A-mediated proliferation and cell cycle progression were c-Myc dependent. Our study provides the role of ACTL6A in cervical oncogenesis and reveals a potential target for therapeutic intervention in this cancer type.

## Introduction

Cervical cancer is the fourth most common malignant tumor in women worldwide, with an estimated 0.604 million new cases and 0.342 million cancer-related deaths (1). It is also the second leading cause of cancer death in women aged 20 to 39 years (2). Although recent global cancer assessments have shown that early and high-quality screening programs have reduced the mortality rate of cervical cancer, premature cervical cancer deaths have risen rapidly in most low- and middle-income countries (3). Current treatment strategies for advanced cervical cancer is a combination of radical surgery, radiotherapy, chemotherapy and immunotherapy, with limited efficacy and potential adverse reactions. The identification of more valuable therapeutic targets can improve the survival of patients with cervical cancer.

Actin-like protein 6A (ACTL6A, also known as BAF53A) is a subunit of the chromatin remodeling complex SWI/SNF that plays a role in the development of neuronal progenitor cells, stem cells and epidermal cells (4, 5). It has been paid more and more attention in the development and progression of various malignant tumors. We reported that ACTL6A is amplification in ovarian cancer and silencing ACTL6A attenuated follicle-stimulating hormone (FSH)-driven glycolysis by downregulation of PGK1 (6). ACTL6A is highly expressed in hepatocellular carcinoma (HCC) tissues and associated with poor prognosis. ACTL6A binds to SOX2 promoter, up-regulates SOX2 expression, and induces epithelial-mesenchymal transition (EMT) of HCC cells through Notch1 pathway (7). ACTL6A also interacts with p63 in head and neck squamous cell carcinoma (HNSCC), and activates the Hippo-YAP pathway to control the transcriptional regulation of proliferation and inhibition of differentiation (8). One recent study reported that ACTL6A could induce resistance to cisplatin treatment (9). These studies suggest that ACTL6A may be a new potential therapeutic target for inhibiting tumor progression. However, it’s biological role in cervical cancer has not been reported.

In this study, we focused on the role of ACTL6A in cervical cancer. We found that the protein level of ACTL6A in cervical cancer tissues was higher than that in adjacent normal tissues. Functional assays confirmed that ACTL6A is an oncogene that promotes the proliferation and cell cycle progression of cervical cancer cells in a c-Myc-dependent manner. Our results reveal the role of ACTL6A in promoting the proliferation of cervical cancer cells and indicate that ACTL6A may be a promising anticancer therapeutic target.

## Materials And Methods

### Reagents

Anti-ACTL6A (ab131272) primary antibodies were obtained from Abcam (Cambridge, USA). Anti-MCM2 (10513-1-AP), anti-Cyclin A2 (18202-1-AP), anti-SKP2 (15010-1-AP) and anti-Tubulin (11224-1-AP) primary antibodies and secondary antibodies were purchased from Proteintech (Wuhan, China).

### Cell Lines and Tissue Specimens

Cervical cancer cell lines Hela and C33A were obtained from the American Type Culture Collection (ATCC, USA). HEK293T was purchased from the Type Culture Collection of the Chinese Academy of Sciences (Shanghai, China). All cells were cultured with DMEM (high glucose, Invitrogen, USA) containing 10% fetal bovine serum (Invitrogen, Carlsbad, CA) and 1% streptomycin/penicillin at 37°C in a humidified atmosphere incubator containing 5% CO_2_.

A total of 124 patients with cervical cancer underwent radical hysterectomy and lymphadenectomy in Shanghai Tenth People’s Hospital from January 2006 to December 2014. All enrolled patients were in the IB1-IIA2 stage. Pathological diagnoses of the samples were performed by two experienced pathologists (Yingze Wei and Jiangtao Xu) basing on the World Health Organization classification in a double-blinded manner. Clinical and pathological characteristics of these samples are shown in **Supplementary Table S1**. This study was approved by the institutional Ethics Committee of Shanghai Tenth People’s Hospital. All patients provided written informed consent.

### Immunohistochemical (IHC) Analysis

IHC analysis of ACTL6A was performed as described earlier (10). The percentages of cells stained were scored as: 0, no positive cells; 1, <25% positive cells; 2, 26%-50% positive cells; 3, 51%-75% positive cells; and 4, >75% positive cells. The staining intensity was scored as: 0, no staining; 1, weak staining (light yellow); 2, moderate staining (yellow brown); and 3, strong staining (brown). The final immunoreactivity scores (IRS) was calculated by percentage of stained cells multiplying staining intensity. The IRS=0 was considered negative expression, IRS<6 was considered low protein level and IRS≥6 was considered high protein level.

### Plasmids, siRNA and shRNA

ACTL6A expression plasmid was kindly provided by Professor Michael D Cole (Geisel School of Medicine at Dartmouth). The E2F1 promoter luciferase reporter plasmid was kindly provided by Dr. Yongbin Yang (Shanghai General Hospital, Shanghai Jiao Tong University). c-Myc was cloned into the pcDNA3.1 vector with Flag-tag at the N terminus by standard cloning method. The following oligonucleotide against genes were used: siRNA/shRNA against ACTL6A-1 (5’-ACCTTACGTTTCATAGCTTTA-3’), siRNA/shRNA against ACTL6A-2 (5’-TGTCAGAGGCACCGTGGAATA-3’), siRNA against c-Myc (5’-CCCAAGGTAGTTATCCTTAAA-3’).

### Real-Time Quantitative PCR (RT-qPCR)

Total RNA was isolated from the cells using RNAiso Plus (9108, Takara) and reverse transcription was performed using PrimeScript™ Reverse Transcriptase (2690S, Takara). All qPCR analyses were performed using SYBR qPCR Master Mix (TOYOBO). Primer sequences are shown in **Supplementary Table S2**.

### Tumor Xenografts

The animal experiment was conducted in accordance with the guidelines of the Institutional Animal Care and Use Committee (IACUC) and the protocol was approved by the ethics committee of Shanghai Tenth People’s Hospital. 5×10^6^ Hela cells infected with lentiviral control shRNA and shACTL6A were subcutaneously injected into the dorsal flank of six-week-old female nude mice (Shanghai SLAC laboratory Animal Co., Ltd). Tumor size was measured every 4 days after 12 days, and the average tumor volume was calculated according to the standard formula: volume = (length × width^2^)/2. On 36th day, the mice were euthanized, tumors were excised and weighed.

### Western Blotting

The transfected cells were extracted using RIPA lysis buffer (150 mM NaCl, 10 mM Tris-HCl pH 7.4, 1 mM EDTA, 1% NP40, 0.1% SDS, 1% sodium deoxycholate, proteinase inhibitor cocktail and phosphatase inhibitor cocktail) for 30 minutes at 4°C and then centrifuged at 12,000 rpm 4°C for 15 minutes, the supernatant was extracted as total protein. The protein sample was mixed with 4×loading buffer, boiled at 100°C for 15 minutes, and the SDS-PAGE gel was used for separation. After that, the proteins on the SDS-PAGE gel were transferred to a nitrocellulose membrane and incubated with the primary antibody at 4°C overnight. Next day, the membrane was incubated with 5% skimmed milk for 45 minutes, and then incubated with secondary antibody for 60 minutes. The immunoblots were detected by using the Odyssey system (LI-COR Biosciences, Lincoln, USA) or the electrochemiluminescence (ECL) imaging system (Tanon, Shanghai, China).

### MTT and Colony-Formation Assay

SiRNA-transfected Hela and C33A cells were seeded into 96-well plates with 1000 cells per well. At different time points (1, 2, 3, 4 or 5 days after plating), 10 μl MTT solution (5 mg/ml final concentration, C0009, Beyotime, China) was added to each well. After incubation at 37°C for 4 hours, 200 μl dimethyl sulfoxide (DMSO) was added, and the color formation was quantified at 490 nm wavelength. In each individual experiment, proliferation under each condition was studied in sextuplicate. For the colony formation experiment, shRNA transfected Hela or C33A cells were seeded into a 12-well plate at a ratio of 1000 cells per well. After 10 days, the cells were fixed with methanol and stained with 0.4% crystal violet. The number of colonies was counted for analysis.

### Cell Cycle Analysis

The cell cycle analysis kit (C1052, Beyotime, China) was used according to the manufacturer’s protocol. At 72 hours after transfection, Hela and C33A cells were harvested by trypsin method and fixed in 70% cold ethanol at -20°C overnight. Then propidium iodide (PI) was stained at 37°C for 30 minutes and the cell cycle distribution was analyzed by flow cytometry with a FACScan flow cytometer (BD Biosciences, USA). The percentage of cells in the G0/G1, S and G2/M phases was analyzed by using FlowJo (version 7.6.2, Tree Star, USA).

### Luciferase Reporter Analysis

HEK293T were inoculated at 5×10^4^ cells per well in 24-well plates. At the same time, 10 ng of Renilla luciferase reporter plasmid and 50 ng of E2F1 promoter luciferase reporter plasmid were transfected. Twenty-four hours after transfection, luciferase activity was measured by using the Dual Luciferase Reporter Gene Kit (Promega, CA, USA), following the manufacturer’s protocol.

### Bioinformatics Analysis

Gene expression data from Cervical Squamous Cell Carcinoma and Endocervical Adenocarcinoma (CESC) available at the TCGA database (TCGA, Provisional) was assessed by using the cBioPortal (http://cbioportal.org) (11, 12). The Oncomine platform (https://www.oncomine.org/) and Gene Expression Omnibus (GEO) datasets were used to compare the expression of ACTL6A in normal and tumor tissues. GSE63514 compared the expression of ACTL6A in 24 normal, 14 cervical intraepithelial neoplasia (CIN) I, 22 CIN II, 40 CIN III and 28 cervical cancers specimens. GSE7803 was used to analyze the expression of ACTL6A between 10 normal squamous cervical epithelia samples and 21 invasive squamous cell carcinomas of the cervix. GSE6791 showed the expression of ACTL6A in 8 cervix uteri samples and 20 cervical cancer samples. GSE7410 evaluated the expression of ACTL6A between 5 non-cervical carcinoma and 19 patients with cervical cancer. GSE9750 compared the expression of ACTL6A in 24 normal cervical epithelium and 33 cervical cancer samples. GSE56303 assessed the expression of ACTL6A in 55 cervical squamous cell carcinomas and 5 cervical adenocarcinomas.

Biological states or processes related to the activities of ACTL6A were evaluated by GSEA v3.0 software (13). Using different gene sets deposited in the GSEA Molecular Signatures Database v6.2 (MSigDB) resource, we identified the differential pathways between the high- and low-ACTL6A expression specimens.

LinkedOmics platform (http://linkedomics.org/login.php) was used to perform GSEA to explore Gene Ontology (GO) biological process, molecular function and KEGG pathways potentially regulated by ACTL6A in cervical cancer (14). The results were demonstrated by ImageGP (http://www.ehbio.com/ImageGP/). LinkedOmics was also used to shown the correlation between the expression of ACTL6A and cell cycle-related genes (CCNA2, MCM2 and SKP2), differentiation genes (S100P, S100A4, KRT7 and TGM2) and Myc target genes (HDAC2, SYNCRIP, GLO1, CDC45, CDK4, PSMD7, MCM4 and XPO1).

### Statistical Analysis

Statistical analysis was performed using GraphPad Prism (Version 6.01, GraphPad Software, Inc., USA). Data were shown as the mean ± SD from at least three independent experiments. The relationship between ACTL6A protein level and clinicopathological features was analyzed by Fisher’s Exact Test and Pearson’s chi-square test. Correlations between the expressions of ACTL6A and related-genes were determined using Spearman’s correlation analysis. Immunoreactivity scores were compared using the Mann-Whitney U test. Student t test was used to calculate the significance of difference between control groups and experimental groups. All tests were two-tailed and *p*-value of less than 0.05 was considered statistically significant.

## Results

### Expression Pattern of ACTL6A in Cervical Cancer

**Figure 1:**
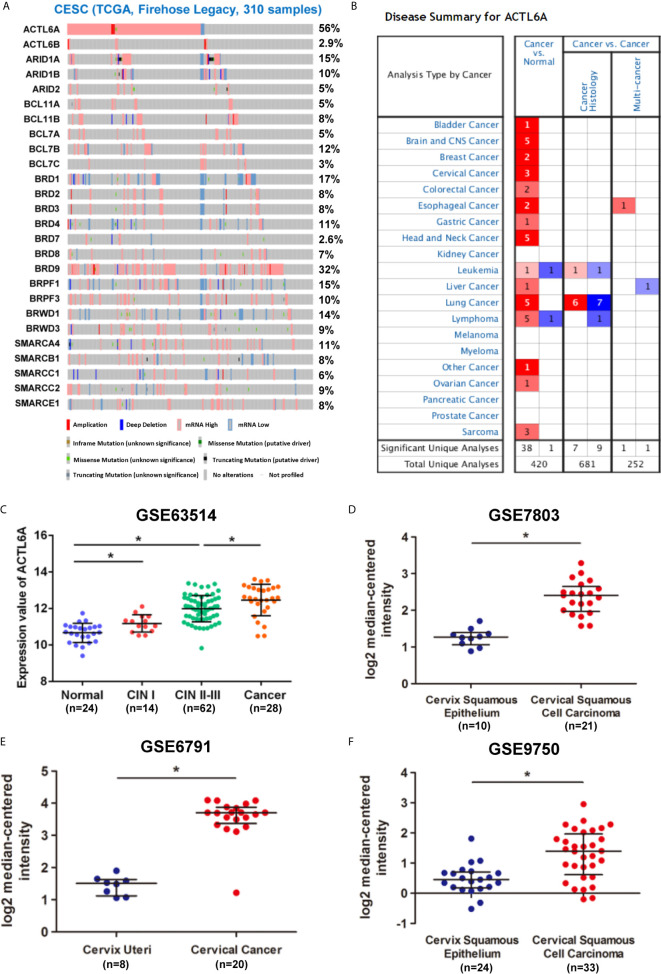
Expression pattern of ACTL6A in cervical cancer. **(A)** Genetic and expression alteration of the subunits of SWI/SNF complex in 310 samples of CESE available at TCGA database by using cBioPortal. **(B)** mRNA expression levels of ACTL6A in various types of cancer from Oncomine. The red or blue intensity is directly proportional to the significance expression level of upregulation or downregulation, respectively. **(C)** Analysis of ACTL6A expression in normal cervix, cervical intra-epithelial neoplasia (CIN) and cervical cancer in GSE63514. **(D–F)** Analysis of ACTL6A expression in normal cervix and cervical cancer in GSE7803, GSE6791 and GSE9750. Error bar = mean ± SD, *p < 0.01.

We first studied the ACTL6A genome and gene expression profile in 310 CESE samples from the TCGA database by using cBioPortal. The mRNA upregulation of ACTL6A and BRD9 was most common in CESC, while changes in other SWI/SNF components were relatively rare (**Figure 1A**). In addition, ACTL6A co-expressed several important genes in the cervical cancer 3q amplicon, including PIK3CA, SOX2 and TP63 (**Supplementary Figure 1A**). Then we analyzed the expression data from the Oncomine and GEO databases. As shown in **Figure 1B**, ACTL6A expression was significantly upregulated in various malignant tumor tissues, including cervical cancer, bladder cancer, breast cancer and esophageal cancer. Compared with the normal cervix, the expression of ACTL6A was higher in CIN and cervical cancer tissues, and the expression of ACTL6A in CIN increased with the rise of the grade (GSE63514, **Figure 1C**). Moreover, ACTL6A expression in multiple data sets of cervical cancer was significantly higher than that of normal cervix (GES7803, GSE6791, GSE7410, GSE9750, **Figures 1D–F**, **Supplementary Figure 1B**). However, there was no difference in ACTL6A between cervical squamous cell carcinoma and adenocarcinoma (GSE56303, **Supplementary Figure 1C**).

**Figure 2:**
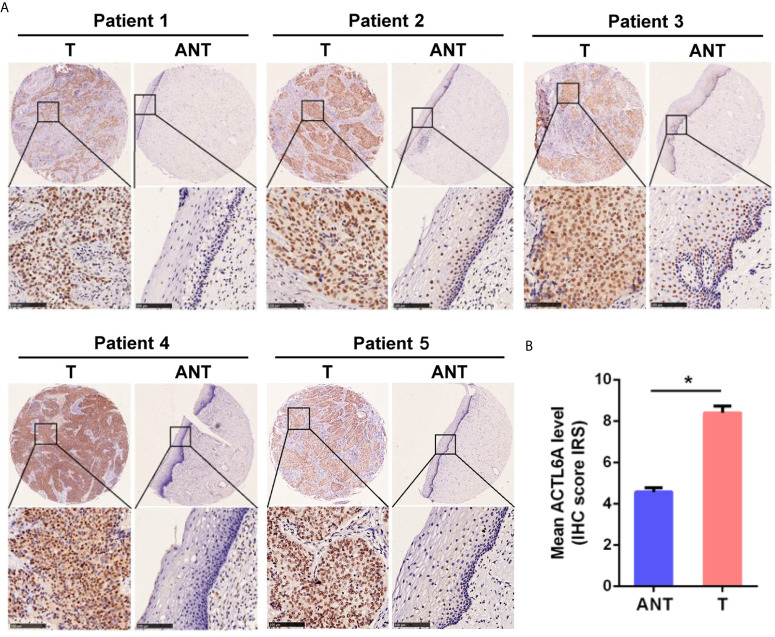
Immunohistochemical analysis of ACTL6A in cervical cancer. **(A)** Representative pictures of cervical cancer tissues and adjacent non-tumor tissues (ANT) stained with an anti-ACTL6A antibody. Scale bar = 100 μm. **(B)** IRS of ACTL6A in cervical cancer and adjacent non-tumor tissues was shown. Error bar = mean ± SD, *p < 0.01.

Then we examined the protein level of ACTL6A in cervical cancer tissue. The cytoplasmic and nuclear immunoreactivity of ACL6A in cervical cancer tissues was significantly higher than that in adjacent nontumor tissues (*p* < 0.01, **Figures 2A, B** **)**. The relationship between ACTL6A and clinicopathological features was also analyzed. According to IRS scores, 124 patients were classified into two groups: low or no rating group (n = 30) and high rating group (n = 94). As shown in **Table 1**, high levels of ACTL6A protein were significantly associated with FIGO stage (*p* < 0.01) and differentiation grades (*p* < 0.01), but not with tumor size (*p* = 0.452), deep stromal invasion (*p* = 0.146) and patient age (*p* = 0.061). These data suggest that ACTL6A amplification may be associated with the progression of cervical cancer.

**Table 1:** Clinical correlations of ACTL6A protein level in cervical cancer.

Variables	ACTL6A protein level		*P*
	Low or none n (%)	High, n (%)	
All case Age (y)	30(24.2)	94(75.8)	
<50	18(32.1)	38(67.9)	0.061^a^
≥50	12(17.6)	56(82.4)	
Differentiation grades			
G1	6(85.7)	1(14.3)	0.001^b^
G2	22(22.2)	77(77.8)	
G3	2(11.1)	16(88.9)	
FIGO stage			
Ib1	19(54.3)	16(45.7)	0.000^b^
Ib2	5(15.2)	28(84.8)	
IIa1	5(13.5)	32(86.5)
IIa2	1(5.6)	18(94.4)
Tumor size (cm)			
≤4	22(26.2)	62(73.8)	0.452^a^
>4	8 (20)	32 (80)	
Deep stromal invasion			
No	14(19.4)	58(80.6)	0.146^a^
Yes	16(30.8)	36(69.2)	

FIGO, International Federation of Gynecology and Obstetrics.

a Pearson Chi-Square.

b Fisher’s Exact Test.

### Correlation Between ACTL6A and Differentiation Genes

**Figure 3:**
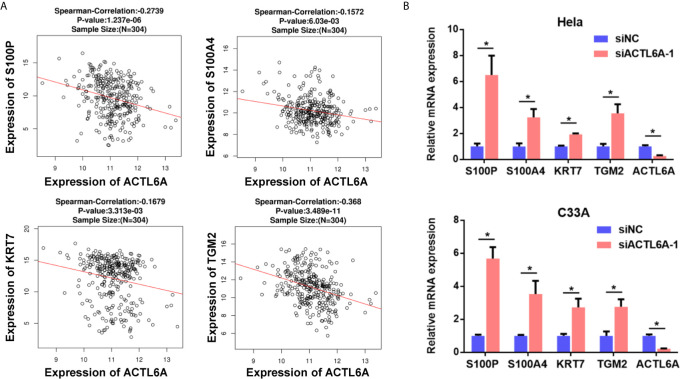
Correlation between ACTL6A and differentiation genes. **(A)** Correlation of ACTL6A mRNA expression with differentiation genes (S100P, S100A4, KRT7 and TGM2) based on TCGA database by using LinkedOmics. *p* value and correlation coefficient were shown. **(B)** The expressions of differentiation genes (S100P, S100A4, KRT7 and TGM2) were detected with RT-qPCR. Each bar represents the mean ± SD of triplicate experiments, *p < 0.01.

One previous study demonstrates that ACTL6A is required to repress differentiation (4). Here, we investigated the correlation between ACTL6A and differentiation genes in cervical cancer samples from TCGA. As shown in **Figure 3A**, the expression of ACLT6A in cervical cancer cohort was negatively correlated with differentiated genes (S100P, S100A4, KRT7 and TGM2). Furthermore, the expressions of S100P, S100A4, KRT7 and TGM2 were also induced by silencing of ACTL6A in cervical cancer cells (**Figure 3B**).

### Role of ACTL6A in Cervical Cancer Cell Proliferation

**Figure 4:**
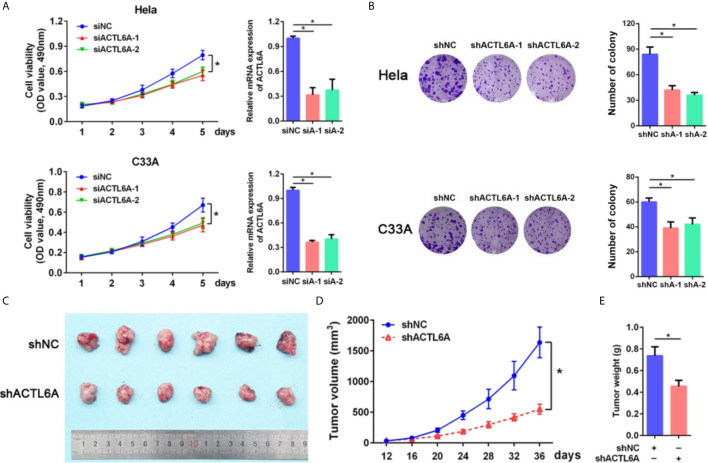
Role of ACTL6A in cervical cancer cell proliferation. **(A)** MTT assay and RT-qPCR analysis of ACTL6A in Hela (upper panel) and C33A (lower panel) cells treated with control (siNC) and ACTL6A siRNAs (siA-1 and siA-2). Each bar represents the mean ± SD of triplicate experiments, *p < 0.01. **(B)** Colony formation assay of Hela (upper panel) and C33A (lower panel) cells transfected with shNC and shACTL6A. Each bar represents the mean ± SD of triplicate experiments, *p < 0.01. **(C)** Representative tumor size in xenograft mouse models bearing with shNC or shACTL6A Hela cells (six per group). Quantifications of tumor growth **(D)** and tumor weight **(E)** from xenograft mouse models were shown. Error bar = mean ± SD, *p < 0.01.

Next, we examined the role of ACTL6A in cervical cancer cell proliferation by using the MTT and colony formation assay. As demonstrated in **Figures 4A, B**, knockdown of ACTL6A reduced the proliferation and colony formation of Hela and C33A cell lines compared with the control group. We also found that the subcutaneous tumors formed by Hela cells transfected with lentiviral shACTL6A were smaller, in both size and weight, than those with shRNA control cells (**Figures 4C** **–E**).

### Identification of Biological Processes and Signaling Pathways

**Figure 5:**
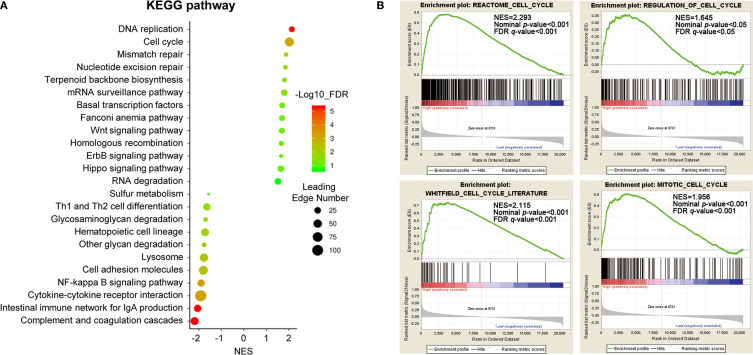
Identification of biological processes and signaling pathways. **(A)** Gene set enrichment analysis showed the correlation between ACTL6A and KEGG pathway in cervical cancer available at LinkedOmics. **(B)** GSEA enrichment plots showed that a series of gene sets including REACTOME CELL CYCLE, REGULATION OF CELL CYCLE, WHITFIELD CELL CYCLE LITERATURE and MITOTIC CELL CYCLE were enriched in the ACTL6A-high group. NES, normalized enrichment score; FDR, false discovery rate.

To gain insight into the potential biological pathways of ACTL6A implicated in the tumorigenesis of cervical cancer, GESA was performed by using Linked Omics platform. The results showed that ACTL6A may be involved in DNA replication, chromosome segregation, mitotic cell cycle phase transitions, cell cycle G2/M and G1/S phase transitions, protein localization of chromosomes, etc. (**Supplementary Figure 2A**). Chromatin DNA binding, nucleosome binding, histone binding, ubiquitin-like protein binding, double-stranded RNA binding and translation initiation factor binding were also potential molecular functions (**Supplementary Figure 2B**). In addition, high expression of ACTL6A was positively correlated with various cancer-related processes of cervical cancer mapping in KEGG pathway (**Figure 5A**). For example, DNA replication, cell cycle, mismatch repair, Wnt signaling pathway and Hippo signaling pathway were closely associated with the expression of ACTL6A in cervical cancer. Moreover, the other four gene sets of cell cycle including REACTOME CELL CYCLE (NES = 2.293, *p* < 0.01), WHITFIELD CELL CYCLE LITERATURE (NES = 2.115, *p* < 0.01), REGULATION OF CELL CYCLE (NES = 1.645, *p* < 0.01) and MITOTIC CELL CYCLE (NES = 1.956, *p* < 0.01) were also proved to be significantly correlated with high expression of ACTL6A (**Figure 5B**).

### Silencing ACTL6A Induces Cell Cycle Arrest in Cervical Cancer Cell

**Figure 6:**
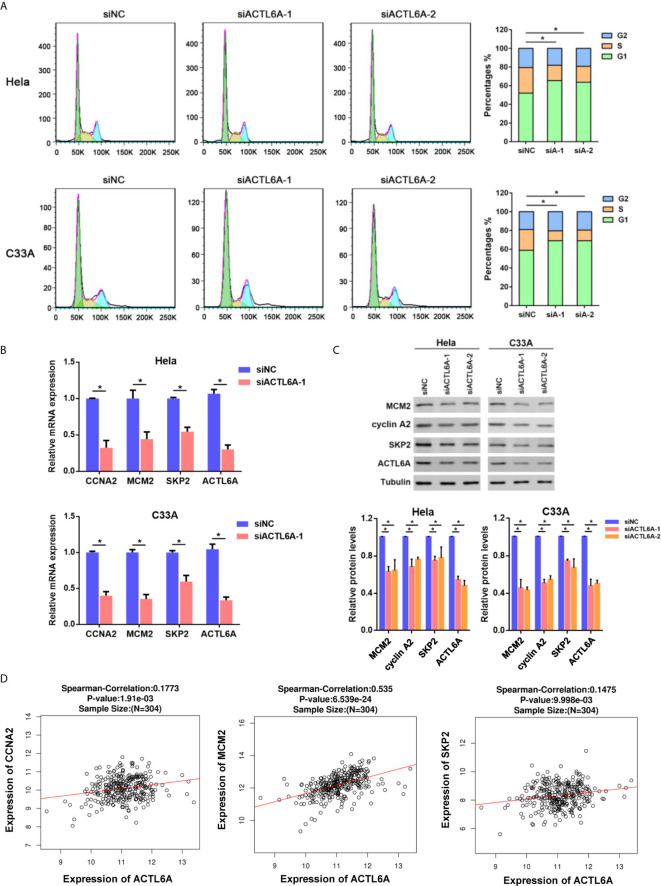
Silencing ACTL6A induces cell cycle arrest in cervical cancer cell. **(A)** Flow cytometric analysis of the cell cycle distribution at 72 hours after treated with siNC or ACTL6A siRNAs in Hela and C33A cells. The percentages of each phase of the cell cycle (G1, S, and G2) are shown. **(B)** The expressions of cell cycle-related genes (MCM2, CCNA2 and SKP2) were detected with RT-qPCR. **(C)** The protein levels of cell cycle-related proteins (MCM2, CCNA2 and SKP2) were detected with Western blotting. **(D)** Correlation of ACTL6A mRNA expression with cell cycle-related genes (MCM2, CCNA2 and SKP2) based on TCGA database by using LinkedOmics. *p* value and correlation coefficient were shown. Each bar represents the mean ± SD of triplicate experiments, *p < 0.01.

As ACTL6A expression was closely associated with cell cycle according to GSEA, we then investigated whether ACTL6A affects the cell cycle of cervical cancer cells. Cell cycle analysis by flow cytometry showed that silencing of ACTL6A significantly increased the percentage of cells in the G1 phase and decreased the proportion of cells in the S phase compared to the control group (**Figure 6A**). Moreover, we evaluated the expression of cell cycle-related genes (CCNA2, MCM2 and SKP2) in cervical cancer cells transfected with siNC or siACTL6A. The mRNA expressions and protein levels of these genes/proteins were decreased in both Hela and C33A cells after knockdown of ACTL6A (**Figures 6B, C** **)**. Correlation analysis also revealed that ACTL6A was positively correlated with these cell cycle regulation genes in clinical samples of cervical cancer from TCGA (**Figure 6D**).

### ACTL6A Induces Cell Cycle Progression *via* c-Myc

**Figure 7:**
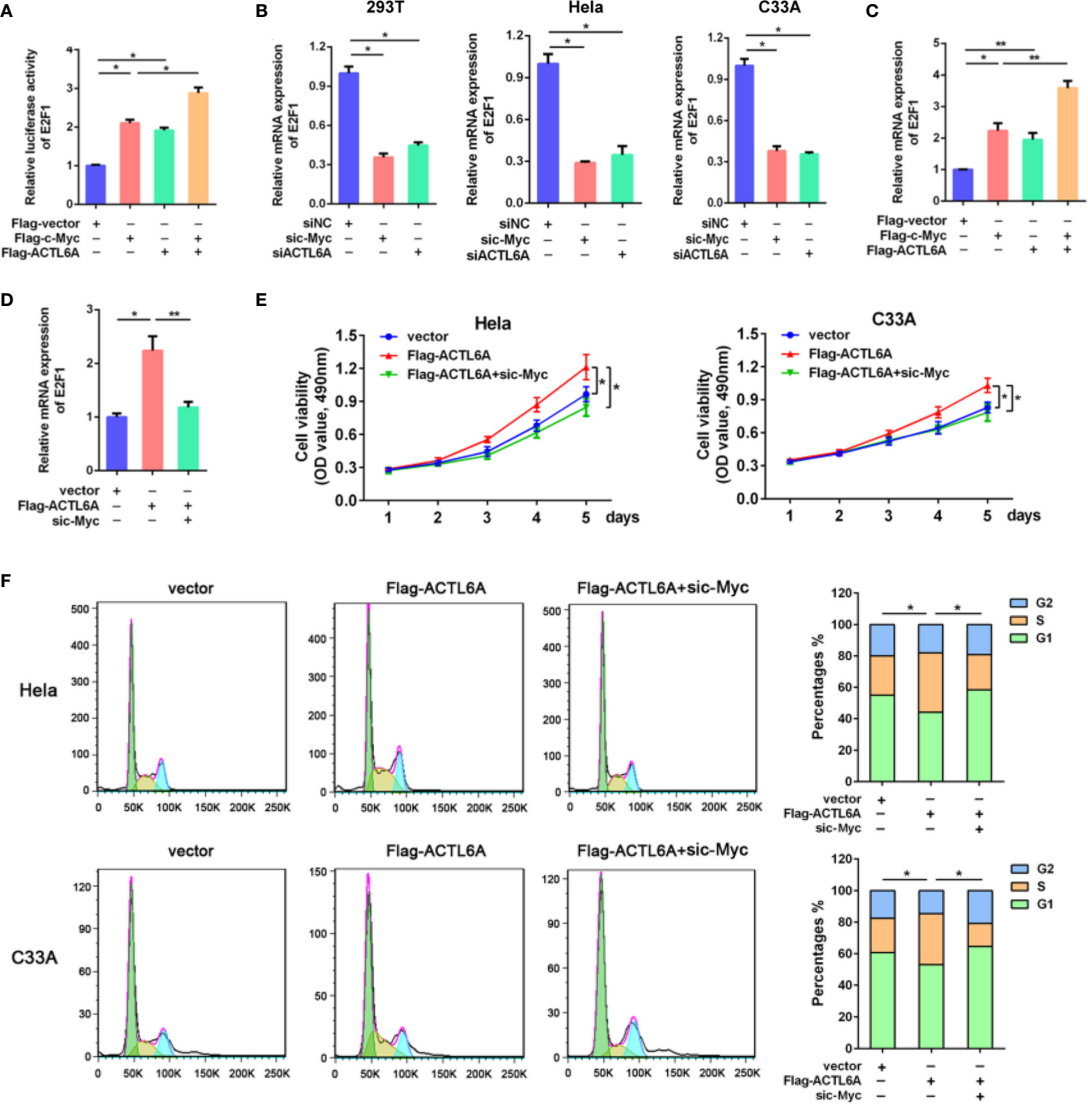
ACTL6A induces cell cycle progression *via* c-Myc. **(A)** E2F1 promoter reporter was transfected with Flag-c-Myc and/or Flag-ACTL6A into HEK293T cells as indicated. Promoter activity was analyzed by Dual-Luciferase assay. **(B)** HEK293T, Hela and C33A cells were transfected with control siNC, ACTL6A siRNA or c-Myc siRNA for 72 hours, then the E2F1 mRNA levels were analyzed using quantitative RT-qPCR. **(C)** Flag-c-Myc and/or Flag-ACTL6A were expressed in HEK293T cells. The E2F1 mRNA levels were analyzed using quantitative RT-qPCR. **(D)** HEK293T cells were transfected with control vector or Flag-ACTL6A or the mixture of Flag-ACTL6A plus the c-Myc siRNA (Flag-ACTL6A + sic-Myc) for 72 hours. The expression of E2F1 was analyzed using quantitative RT-qPCR. **(E)** MTT assay results. Hela and C33A cells were transfected with control vector or Flag-ACTL6A or the mixture of Flag-ACTL6A plus the c-Myc siRNA (Flag-ACTL6A + sic-Myc). **(F)** Flow cytometric analysis of the cell cycle distribution at 72 hours after treated with control vector or Flag-ACTL6A or the mixture of Flag-ACTL6A plus the c-Myc siRNA (Flag-ACTL6A + sic-Myc). The percentages of each phase of the cell cycle (G1, S, and G2) are shown. Each bar represents the mean ± SD of triplicate experiments, *p < 0.01, **p < 0.05.

To further investigate the molecular mechanism of ACTL6A-mediated cell cycle, GSEA showed that the gene set HALLMARK_MYC_TARGETS was positively correlated with the high expression of ACTL6A (**Supplementary Figure 3A**). In addition, the online analysis tool of the string database of the protein-protein interaction (PPI) network revealed that ACTL6A may form a complex with c-Myc and its dimerization partner Max (**Supplementary Figure 3B**). Then we evaluated the effect of ACTL6A on c-Myc dependent transfection of the well-defined c-Myc target gene E2F1 (15). HEK293T cells transfected with either ACTL6A or c-Myc plasmid and E2F1 promoter. We observed that both c-Myc and ACTL6A activated the E2F1 promoter, while overexpression of c-Myc and ACTL6A further stimulated E2F1 promoter activity (**Figure 7A**). In addition, silencing of either c-Myc or ACTL6A decreased E2F1 expression (**Figure 7B**), while overexpression of both c-Myc and ACTL6A resulted in increased E2F1 mRNA expression (**Figure 7C**). Importantly, c-Myc knockdown blocked ACTL6A-induced E2F1 (**Figure 7D**). Moreover, we demonstrated that the expression of ACTL6A was significantly correlated with c-Myc target genes, such as HDAC2, CDC45, CDK4, PSMD7 and MCM4 (**Supplementary Figure 3E**).

To further verify the cell growth-promoting function of ACTL6A is mediated by c-Myc, we showed that overexpression of ACTL6A promoted the proliferation of Hela and C33A cells, while knockdown of c-Myc significantly inhibited ACTL6A-increasd proliferation (**Figure 7E**). Flow cytometry analysis also demonstrated that ACTL6A-induced cell cycle progression was abolished by knockdown of c-Myc (**Figure 7F**). These results indicated that ACTL6A regulates the proliferation and cell cycle progression of cervical cancer cells through c-Myc.

## Discussion

Increasing evidences have suggested that ACTL6A plays important roles in the carcinogenesis processes. However, the pathological role of ACTL6A was remained poorly understood in cervical cancer. Here, we reported a significant increase in ACTL6A protein levels in cervical cancer tissues. Our study suggested that ACTL6A promoted cell proliferation through regulation of cell cycle *via* c-Myc in cervical cancer. We suggest that ACTL6A plays an oncogenetic role in the development of cervical cancer and has potential clinical values in targeted therapy.

The SWI/SNF complex was originally described as a multi-subunit protein complex that controls gene expression and various important signaling pathways (16). Mutations, deletions and translocations involving SWI/SNF complex subunits occur frequently in cancer (17–19). In contrast, some studies have reported that ACTL6A amplification and overexpression are common events in cancers (6–9, 20–25). Consistent with these studies, we found an increase in ACTL6A in cervical cancer by combining the CESC genome and expression profile based on multiple public database and IHC detection in cervical cancer tissues. In addition, by comparing different CIN stages, we found that the expression of ACTL6A increased from low-level CIN to pre-invasive CIN lesions. However, other subunits of the SWI/SNF complex have relatively little genetic alterations in CESC. This supports the idea that ACTL6A is a special oncogenic subunit in the SWI/SNF complex in cervical cancer. In addition, one previous study revealed that depletion of ACTL6A in lysates from HNSCC cells could induce quantitative depletion of other SWI/SNF complex subunits, confirming the binding of ACTL6A with an intact SWI/SNF complex (8). In that case, more evidences for the assembly status of SWI/SNF components upon ACTL6A silencing might help to reveal the role of ACTL6A in more detail.

Previous studies have shown that ACTL6A has carcinogenic activity and acts as an inducer of EMT (7, 25). In epidermal squamous cell carcinoma (SCC), ACTL6A has been proven to interact with p53 DNA response elements in the gene promoter of p21^Cip1^ and maintain the aggressive phenotype (22). ACTL6A is also co-localized and interacts with Sox2 and p53, and inhibits differentiation in leukemia cells (21). Moreover, ACTL6A enhances malignant behaviors of glioma cells by increasing the stability and abundance of YAP/TAZ protein (20). It also interacts with p63, promoting cell proliferation and inhibiting differentiation of HNSCC through Hippo-YAP pathway (8). Notably, although it has been reported that knockdown of ACTL6A inhibits HPV E6/E7 oncogene expression and restores the p53-related signaling pathway in cervical cancer cells (26), its biological roles in cervical cancer remain largely unknown. In current study, we provide evidence that ACTL6A promotes the proliferation, clone formation and *in vivo* tumor formation of cervical cancer cells. GSEA analysis showed that the increased expression of ACTL6A was related to biological processes such as cell cycle and DNA replication. In fact, we found that silencing ACTL6A increases the percentage of G1 phase cells and decreased the expression of cell cycle related genes. These results are similar to some previous studies (4, 24), which further confirmed that ACTL6A can promote the occurrence of different malignancy through regulating cell cycle progression.

We also provided data on the potential mechanism by which ACTL6A promotes cell cycle progression. C-Myc is an oncogenic transcription factor, which is frequently deregulated in cancers and is associated with malignant progression (27). It is now clear that c-Myc is closely related to the cell cycle process, and the molecular mechanism by which c-Myc stimulates the cell cycle has been well elucidating (28). Previous studies showed that ACTL6A can form a complex with c-Myc, which is critical for the oncogenic activity of c-Myc (29), and ACTL6A also promotes the recruitment of Myc on CDK2 promoters (23). The role of ACTL6A-stimulated c-Myc activity in cervical cancer was demonstrated here. We found that co-expression of ACTL6A enhanced the activity of E2F1 promoter induced by c-Myc, while knockdown of c-Myc reduced the activity and expression of E2F1 stimulated by ACTL6A. The expression of ACTL6A was also significantly correlated with c-Myc target genes. Moreover, the functions of ACTL6A to promote cell growth and induce cell cycle progression is dependent on c-Myc. These findings confirm an important role of ACTL6A in cervical cancer by influencing c-Myc-driven tumorigenesis. Nevertheless, more researches are needed to reveal the mechanism in greater detail.

## Conclusion

In summary, the present study showed that overexpression of ACTL6A was significantly associated with malignant behavior of cervical cancer. Mechanistic data revealed that ACTL6A promoted tumor growth and cell cycle progress by regulating c-Myc activity. Therefore, inhibition of ACTL6A might be a promising potential therapeutic strategy for cervical cancer.

## Data Availability Statement

The original contributions presented in the study are included in the article/**Supplementary Material**. Further inquiries can be directed to the corresponding authors.

## Ethics Statement

The study was reviewed and approved by the Ethics Committee of Shanghai Tenth People’s Hospital of Tongji University. The patients/participants provided their written informed consent to participate in this study. The study was reviewed and approved by the Ethics Committee of Shanghai Tenth People’s Hospital of Tongji University (SHDSYY-2018-2578).

## Author Contributions

JWZ and ZPC contributed to the study conception and design. QYW, ZZC and YZW performed the study and analyzed the data. The draft of the manuscript was written by QYW and JWZ. All authors discussed the results and agreed to be accountable for all aspects of the work. All authors contributed to the article and approved the submitted version.

## Funding

The study was supported by Grants from the National Natural Science Foundation of China (81802589 and 81502230).

## Conflict of Interest

The authors declare that the research was conducted in the absence of any commercial or financial relationships that could be construed as a potential conflict of interest.

## Publisher’s Note

All claims expressed in this article are solely those of the authors and do not necessarily represent those of their affiliated organizations, or those of the publisher, the editors and the reviewers. Any product that may be evaluated in this article, or claim that may be made by its manufacturer, is not guaranteed or endorsed by the publisher.

## Supplementary Material

Supplementary Figure 1 **(A)** Genetic alterations of ACTL6A, PIK3CA, SOX2 and TP63 in 310 samples of CESE available at TCGA database by using cBioPortal. **(B)** Analysis of ACTL6A expression in normal cervix and cervical cancer in GSE7410. **(C)** Analysis of ACTL6A expression in cervical squamous carcinomas and adenocarcinomas in GSE56303. Error bar = mean ± SD, **p* < 0.01.

Supplementary Figure 2 Gene set enrichment analysis showed the correlation between ACTL6A and GO biological process **(A)** and GO molecular function **(B)** in cervical cancer available at LinkedOmics.

Supplementary Figure 3 **(A)** GSEA enrichment plot showed that the HALLMARK MYC TARGETS gene set was enriched in the ACTL6A-high group. **(B)** STRING database analysis of the PPI network for ACTL6A and c-Myc. **(C)** The knockdown or over-expression transfection efficiency of ACTL6A and c-Myc detected by Western blots. **(D)** The knockdown transfection efficiency of ACTL6A and c-Myc detected by RT-PCR. Error bar = mean ± SD, **p* < 0.01. **(E)** Correlation of ACTL6A mRNA expression with c-Myc target genes (HDAC2, SYNCRIP, GLO1, CDC45, CDK4, PSMD7, MCM4 and XPO1) based on TCGA database by using LinkedOmics. *p* value and correlation coefficient were shown.
