# Supplementary figures and images for: Potential Role of SWI/SNF Complex Subunit Actin-Like Protein 6A in Cervical Cancer
Source: Front Oncol. 2021 Jul 29;11:724832. doi: 10.3389/fonc.2021.724832 (PMC8358818; doi:10.3389/fonc.2021.724832)

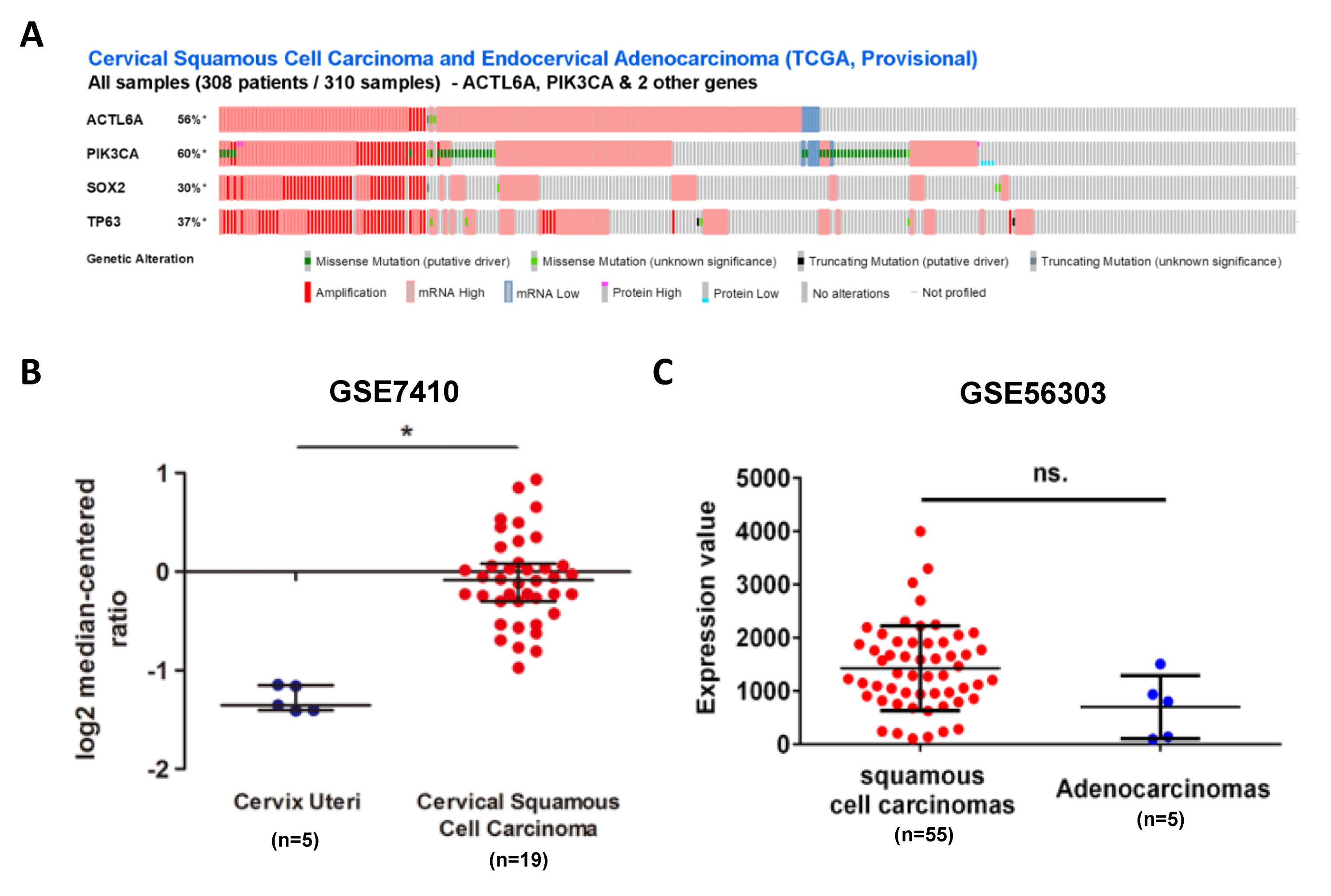

Supplement: Supplementary Figure 1 — (A) Genetic alterations of ACTL6A, PIK3CA, SOX2 and TP63 in 310 samples of CESE available at TCGA database by using cBioPortal. (B) Analysis of ACTL6A expression in normal cervix and cervical cancer in GSE7410. (C) Analysis of ACTL6A expression in cervical squamous carcinomas and adenocarcinomas in GSE56303. Error bar = mean ± SD, *p < 0.01. [file Image_1.tif]

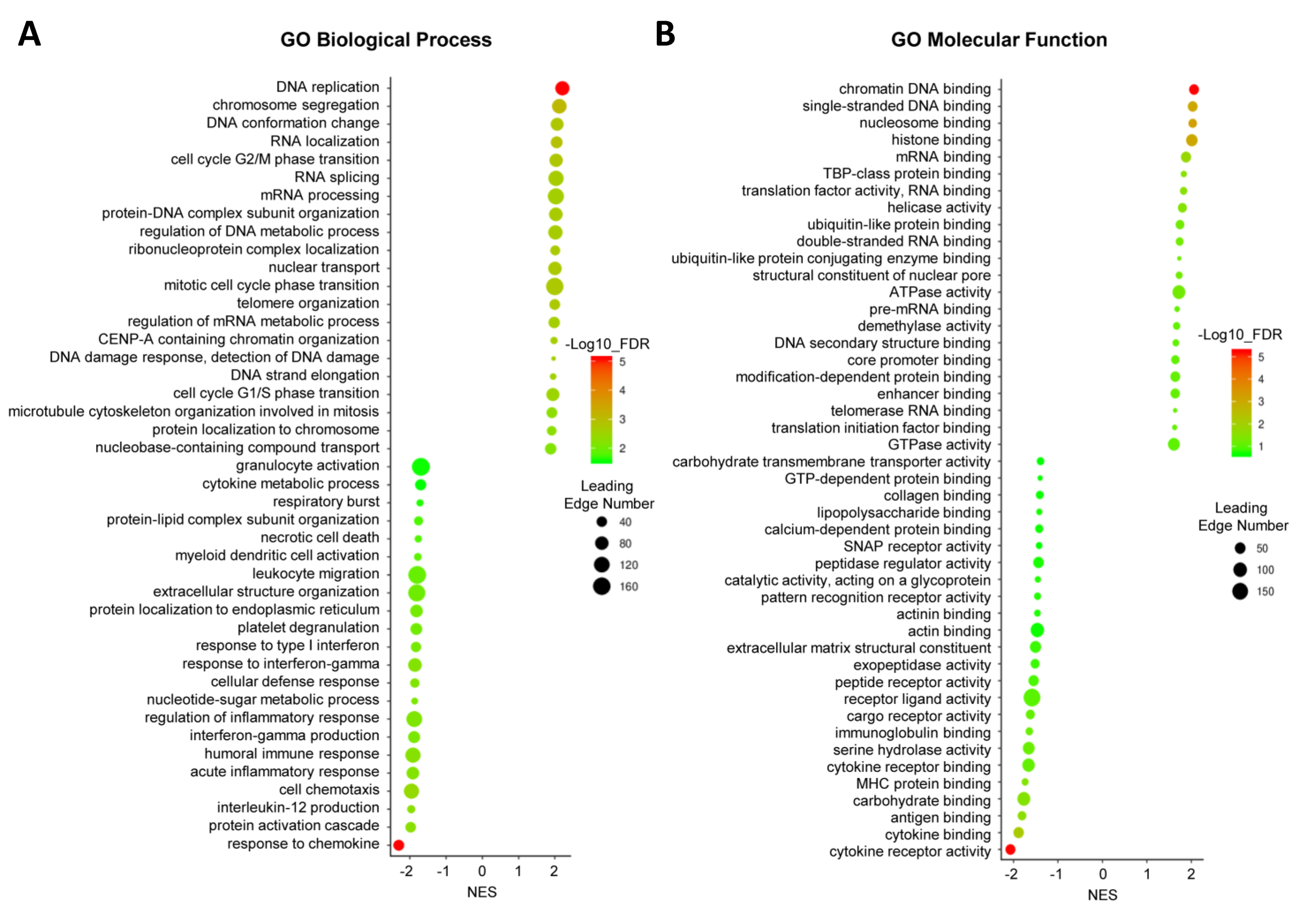

Supplement: Supplementary Figure 2 — Gene set enrichment analysis showed the correlation between ACTL6A and GO biological process (A) and GO molecular function (B) in cervical cancer available at LinkedOmics. [file Image_2.tif]

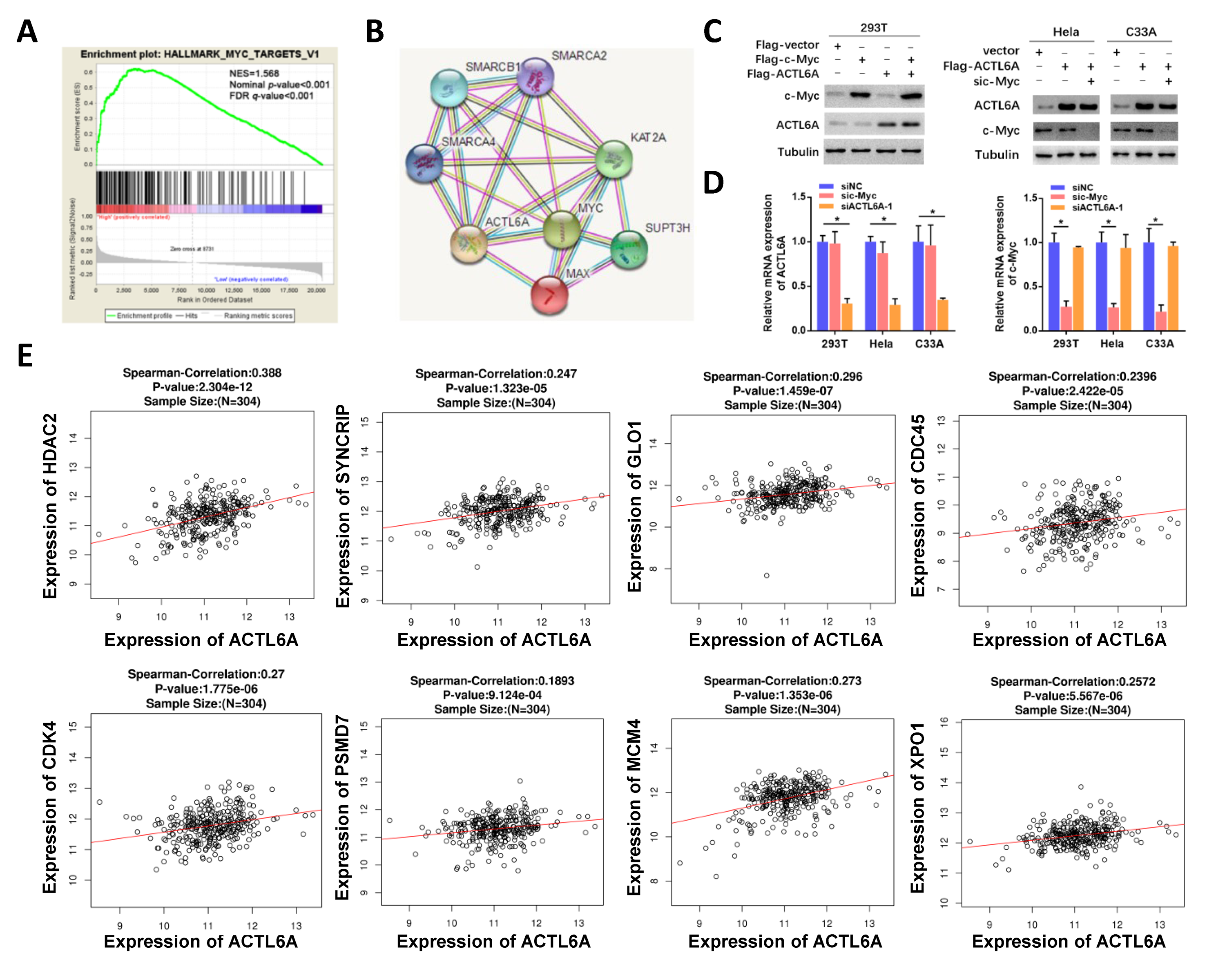

Supplement: Supplementary Figure 3 — (A) GSEA enrichment plot showed that the HALLMARK MYC TARGETS gene set was enriched in the ACTL6A-high group. (B) STRING database analysis of the PPI network for ACTL6A and c-Myc. (C) The knockdown or over-expression transfection efficiency of ACTL6A and c-Myc detected by Western blots. (D) The knockdown transfection efficiency of ACTL6A and c-Myc detected by RT-PCR. Error bar = mean ± SD, *p < 0.01. (E) Correlation of ACTL6A mRNA expression with c-Myc target genes (HDAC2, SYNCRIP, GLO1, CDC45, CDK4, PSMD7, MCM4 and XPO1) based on TCGA database by using LinkedOmics. p value and correlation coefficient were shown. [file Image_3.tif]
