# Supplementary material for: Potential Role of SWI/SNF Complex Subunit Actin-Like Protein 6A in Cervical Cancer
Source: Front Oncol. 2021 Jul 29;11:724832. doi: 10.3389/fonc.2021.724832 (PMC8358818; doi:10.3389/fonc.2021.724832)
Supplement: Supplementary file 4 [file Table_1.docx]

Supplementary Table S1. Clinical and pathological characteristics of the samples analyzed in this study (n=124)

| Clinical/pathological  characteristics | No. of Patients  （n） |
| --- | --- |
|  |  |
| All case  Age (y) | 124 |
| <50 | 56 |
| ≥50 | 68 |
| Differentiation grades |  |
| G1 | 7 |
| G2 | 99 |
| G3 | 18 |
| FIGO stage |  |
| Ib1 | 35 |
| Ib2 | 33 |
| IIa1  IIa2 | 37  19 |
| Tumor size (cm) |  |
| ≤4 | 84 |
| >4 | 40 |
| Deep stromal invasion |  |
| No | 72 |
| Yes | 52 |

G1, well differentiated; G2, moderately differentiated; G3, poorly differentiated; FIGO, International Federation of Gynecology and Obstertrics.
