# Supplementary material for: Potential Role of SWI/SNF Complex Subunit Actin-Like Protein 6A in Cervical Cancer
Source: Front Oncol. 2021 Jul 29;11:724832. doi: 10.3389/fonc.2021.724832 (PMC8358818; doi:10.3389/fonc.2021.724832)
Supplement: Supplementary file 5 [file Table_2.docx]

Supplementary Table S2. Primer sequences used in this study.

| gene | primer |
| --- | --- |
| ACTL6A | Forward 5’-CTATGCAGTGCAGAGAACTCTTC-3’ |
|  | Reverse 5’-CCTCGTAACCTGAGGCAACTT-3’ |
| CCNA2 | Forward 5’-GGATGGTAGTTTTGAGTCACCAC-3’ |
|  | Reverse 5’-CACGAGGATAGCTCTCATACTGT-3’ |
| SKP2 | Forward 5’-ATGCCCCAATCTTGTCCATCT-3’ |
|  | Reverse 5’-CACCGACTGAGTGATAGGTGT-3’ |
| MCM2 | Forward 5’-ATGATCGAGAGCATCGAGAACC-3’ |
|  | Reverse 5’-GCCAAGTCCTCATAGTTCACCA-3’ |
| E2F1 | Forward 5’-CATCCCAGGAGGTCACTTCTG-3’ |
|  | Reverse 5’-GACAACAGCGGTTCTTGCTC-3’ |
| S100P | Forward 5’-AAGGATGCCGTGGATAAATTGC-3’ |
|  | Reverse 5’-ACACGATGAACTCACTGAAGTC-3’ |
| S100A4 | Forward 5’-GATGAGCAACTTGGACAGCAA-3’ |
|  | Reverse 5’-CTGGGCTGCTTATCTGGGAAG-3’ |
| KRT7 | Forward 5’-TCCGCGAGGTCACCATTAAC-3’ |
|  | Reverse 5’-GCTCTGTCAACTCCGTCTCAT-3’ |
| TGM2 | Forward 5’-CGTGACCAACTACAACTCGG-3’ |
|  | Reverse 5’-CATCCACGACTCCACCCAG-3’ |
| β-actin | Forward 5’-CATGTACGTTGCTATCCAGGC-3’ |
|  | Reverse 5’-CTCCTTAATGTCACGCACGAT-3’ |
